# The Emergence of Endophytic Microbes and Their Biological Promise

**DOI:** 10.3390/jof4020057

**Published:** 2018-05-16

**Authors:** Gary Strobel

**Affiliations:** Department of Plant Sciences, Montana State University, Bozeman, MT 59717, USA; uplgs@montana.edu

**Keywords:** fungi, endophytes, natural products, plant microbiome, mycodiesel

## Abstract

As is true with animal species, plants also have an associated microflora including endophytes as well as microbes associated with the phyllosphere and rhizosphere (plant surfaces) and this is considered the plant microbiome. However, those organisms within virtually all tissues and organs of the plant are known as endophytes. Most often fungi are the most frequently recovered endophytes from plant tissues, but bacterial forms generally occur in greater numbers, but not in species varieties. The exact biological/biochemical role of the endophyte in the plant and how it interacts with the plant and other endophytes and plant associated organisms has not been intensely and carefully examined. However, this has not stopped investigators in exploring the direct utility of endophytes in boosting plant production, and discovering that endophytes can directly influence the plant to resist temperature extremes, drought, as well as the presence of disease causing organisms. Also, because of the relationships that endophytes seem to have with their host plants, they make a myriad of biologically active compounds some of which are classified as antibiotics, antioxidants, anticancer agents, volatile antimicrobial agents, immunosuppressive compounds, plant growth promoting agents, and insecticides. These endophytic compounds represent a wide range of organic molecules including terpenoids, peptides, carbohydrates, aromatics, hydrocarbons and others and it seems that these compounds may have a role in the host microbe relationship. Most recently and quite surprisingly, some endophytes have been discovered that make hydrocarbons of the types found in diesel and gasoline fuels. In addition, recently discovered are epigenetic factors relating to the biology and biochemistry of endophytes. Interestingly, only about 1–2% of the entire spectrum of 300,000 known plants have been studied for their endophyte composition. Additionally, only a few plants have ever been completely studied including all tissues for the microbes within them. Likewise, the vast majority of plants, including those in oceans and lower plant forms, have never been examined for their endophytes. Furthermore, endophytes representing the “microbiome” of world’s major food plants as they exist in their native “centers of origin” are largely unknown. This non-classical review is intended to provide background information on aspects of developments in endophyte biology and more importantly the identification of new questions in this field that need to be addressed. The review is primarily based on the author’s long held experience in this field.

## 1. Introduction

Endophyte biology is an emerging field. With many new developments in natural product characterization, along with their increasing use in biological control and the application of these organisms to sustain and assist crop production, the field has recently seen a huge jump in scientific attention and interest which has sprung from approaches and methods used in classical plant pathology [1]. These organisms are obtained from all types of plant tissues that seemed to bear no external evidence for the presence of any life forms within them. The advent of important and novel strides being made in the make-up and subsequently the importance of the human microbiome has sparked a huge interest in the plant microbiome (endophytes) and how these organisms may influence the development and, the ability of a plant to resist disease, drought, heat, cold and other insults [2]. In addition, there has been renewed interest in endophytes since many of them have been shown to produce important compounds of pharmaceutical and commercial interest [3]. This list includes novel anticancer agents, antibiotics, novel immunosuppressive compounds, volatile antibiotic mixtures, antioxidants and most recently—fuel related hydrocarbons [3,4]. It appears that the field is ripe for scientific discovery and invention in a number of important scientific areas.

Since the early times, the definition of an endophyte has more or less remained the same—“a microorganism associated with living plant tissues that produces no apparent indication of its presence in the plant and seems not to cause harm to the host [5].” Endophytes have been isolated from virtually all plant organs (roots, stems, leaves, flowers, fruits, and seeds) and the most commonly observed endophytes are the fungi (usually Ascomycteous fungi associated with Fungi Imperfecti), as well as bacteria and this includes the filamentous bacteria which are the Actinomycetes. Periodically, a Zygomycete, and a Basidiomycete and are also isolated. In the future, it may be the case that other life forms may be found that can be considered endophytes such as the mycoplasmas. There is some certainty that all plant forms, including those in the world’s oceans, are hosts or are potential hosts for one or more endophytes [6]. Additionally, many of the lower plant forms such as mosses and liverworts are also hosts to endophytes [7]. This review will primarily focus on fungal endophytes of higher plant forms.

As with most biological sciences, the initial work on endophytes began as an observational science with investigators who were keenly interested in isolating, identifying every possible endophyte from a given plant and then moving on to the next plant species [5,8]. Much of this early work was being done by the Petrini’s and their group in Switzerland [9]. However, in spite of all of the reports on endophytes from Europe, Canada and the USA, it appears that hundreds, if not thousands of novel endophytic species still remain to be discovered especially in those remote, unique and untouched forests, fields and even oceans of the world. Thus, recent efforts in India, Thailand, Brazil and China have resulted in a plethora of new information on fungal endophytes of tropical plant species. In addition, investigators, at the ecological level, have begun to study how endophytes may influence the biology of the plant enabling it to resist biological as well as environment stresses [2,10,11,12]. Collectively, these efforts have crystallized thinking about these organisms and how they represent the “plant microbiome.” This switch in thinking from endophytes as strictly an academic subject to that of practical biology now has the potential for crop associated endophytes to be useful biological tools in promoting plant growth and assisting the plant to better cope with environmental and biological stresses.

On another note, studies on the distribution of endophytes quickly led to some attention be paid to the relationship of endophytes to their host plants. It has been learned that endophytes are mutualists and some, on occasion may become pathogenic when environmental and physiological conditions of the plant may allow [5,8]. However, the exact nature of the interaction with the plant and the products and processes involved in the interactions has gone missing from the literature. What has appeared however is conjecture on what might be happening in the microbe-plant interaction based on tangential observations and speculation.

The likelihood of finding taxonomic novel endophytic microbes is the greatest in the world’s tropical and temperate rainforests and also with plants growing in peculiar and unexpected places such as harsh deserts, high alpine areas, or even in the world’s oceans [3]. It is to be noted that novel endophytes probably do not commonly exist in botanical gardens in population dense areas because of the pollutants in the air that can and do affect microbial growth and activity. The same can be said of unusual plants artificially placed on university campuses where the same nasty pollution elements undoubtedly also exist and researchers can find easy access to plant material but take no thought of the microbes. Ultimately, with taxonomic novelty, it seems, the chances of finding chemical novelty greatly increases [3]. In addition, with the discovery of novel organisms possessing unique biochemical properties there are opportunities for the development of uses of such organisms. This necessarily implies the extreme likelihood for patenting possibilities for new organism and new product development.

This review is certainly not intended to cover all aspects and all discoveries related to fungal endophytes, but it will cover the main recent focal points in this field from the perspective of work done in the author’s own laboratory. Then, as part of the content of each focal point area the author has put forth his ideas and suggestions on what he sees as the potential for future studies in the given area and provides suggestions and guidance on how work on endophytes should be encouraged in some areas and discouraged in others. Specific examples that are given are used to illustrate important considerations when working with endophytes, their discovery, their biology, and their utility. Also discussed and referenced are certain critical techniques that apply to the science of endophyte biology and how they may be useful and applicable to those working in this field.

## 2. Endophytes, Isolation, Storage, Fundamental Biology

Many of the commonly isolated fungal genera associated with plants as pathogens can also be isolated as fungal endophytes and upon inoculation of the plant with the endophyte one does not witness the development of disease symptoms. Some of these fungal organisms may represent new endophytic species or biotypes. Some of the commonly fungal genera that we have commonly isolated include *Fusarium* sp., *Colletotrichum* sp., *Phoma* sp., *Pestalotiopsis* sp., *Xylaria* sp., *Cladosporium* sp., *Curvularia* sp., and a host of others. The most common represented group of fungi isolated as endophytes are the Fungi Imperfecti whose perfect stage is commonly the Ascomycetes. Less frequently isolated members of the groups Basidiomycetes and Zygomycetes.

The methods of isolating these organisms are well known and usually involve the use of a surface treatment solution to rid the selected plant material of unwanted contaminants. This usually involves a thorough rinse in a 70% ethanolic solution followed by flaming the tissue or treatment with a Clorox solution. One must be careful as to not over treat the tissue to kill or harm the endophytes associated with epidermal tissues. Plant tissues are placed on water agar or other agars that may represent selective media containing certain antibiotics to discourage bacterial growth. Over the years we have realized that plant associated mites can cause major damage to endophyte based experimentation in the laboratory and can easily be handled by adding a small crystal or two taken from a common moth ball and placed into a plastic box containing Petri plates from which the endophytes will eventually be selected (Strobel–unpublished, 2018). Mite contamination of the laboratory can literally halt all scientific progress on endophytes until it is cleaned up. Once obtained in pure culture, we have learned that most tropical endophytes may be best preserved for future work by growing them on doubly autoclaved barley seed (hydrated), placed directly in small freezer vials and stored at −70 °C [13].

In order to do taxonomic work on novel fungi it is important to have one or more spore stages of the organism available for description. Frequently endophytes show no sporulation on potato dextrose agar. While a choice of other agars may provide some help in encouraging sporulation, the best approach is to use gamma irradiated carnation leaves placed on water agar as a medium of choice [14]. In addition, as an alternative one may use pieces of the plant host material that has been autoclaved on a water medium that may encourage fructification of the organism. One could also even make a host based concoction agar medium from stem, leaf or root materials and eventually use more purified plant compounds that are critical for fructification [15]. Ideally, one can use the fungus growing and fructifying on these media as a source of biological material for scanning electron and light microscopy as aids in identifying the organism. Limited molecular techniques such sequencing targeting the ITS region are an important aid in identification and should be used to assist, but not used exclusively in trying to describe an organism. When no spore forms are evident under a multitude of growth conditions, one must rely on a host of other methods to assist in identification and description at the taxonomic level. This includes a complement of molecular gene sequence information starting at the ITS region and moving to specifically targeted gene sequence information involving certain enzymes, other analytical chemical data entries including an analysis of volatile chemicals, and scanning electron microscopy of any unique hyphal/spore/fruiting body characteristics. Ultimately, any data gathered must be placed in context with data that exists in the mycological literature for other already known and previously described fungal species. It is to be noted that one must be prepared to face the taxonomic literature and scrutiny of the mycological community when working in the field of endophyte biology especially if one is intent on finding and thence describing novel microbes.

Microorganisms requiring plant tissues to complete their life cycle are classified as “obligate.” Well-documented examples of obligate endophytes are found among mycorrhizal fungi and members of the fungal genera Balansia, Epichloë, and Neotyphodium, from the family Clavicipitaceae (Ascomycota). Interestingly, there is little work demonstrating if there are other obligate endophytes, that are not specifically mycorrhizal fungi or members of the Clavicipitaceae. It is generally suspected that they do exist based on what is known and understood from obligate plant pathogens such as the rust and powdery mildew causing fungi and assuming that the number of host species is constant, the total number of host specific endophytic species can be extrapolated from the number of plant species [16]. Metagenomic analyses for the total population of endophytes in plants is a challenge since there is the technical limitation of separating fungal from host DNA. Basically, the plant DNA is much more abundant than fungal DNA which confounds the analysis. Additionally, we have observed that frequently slow growing unidentified endophytes do arise from many plant species. Upon the second transfer to a potato dextrose medium they again begin to grow very slowly and then gradually die as if some critically important nutrient was missing from the medium. Obviously, further work on such organisms is confounded by absence of the organism. We have seen hints of this phenomenon over the years in my laboratory but lack of available host plants (since many were obtained in the world’s jungles) to substantiate the initial observations made further work on the problem virtually impossible to study.

Also needed are studies showing the complete endophyte characterization of a given plant species. This should include mention of all organisms associated with all tissues and organs of the plant including flowers, fruits and roots which are sometimes difficult specimens to find and study. It is known that the make-up of the population of microbes in a plant will vary as a function of season, the organs being studied, and the host itself. It appears, however, that it is exceedingly interesting to find, identify, and describe totally novel fungal genera and species that are acting as endophytes. The biological potential of such studies is great given the fact, as previously mentioned, that novel taxonomy portends the presence of novel chemistry.

Specifically, I do not completely encourage studies describing the endophytes of a previously unstudied plant that show no novelty in the endophytic composition of the plant, any novel organism or other unique scientific contribution resulting from the study since so little new is to be learned. On the other hand, if a novel fungal genus is isolated from the plant then it is worthwhile to do the work that properly describes the new organism. In addition, if new uses and concepts are found with already known organisms this too can be valuable information. Furthermore, it must be recognized that the use of limited molecular genetic techniques to assist in the identification of the endophytic flora of a plant can be helpful but misleading if no attempts are made at the actual isolation and examination of the host endophytes. It is well understood that identification of fungi at the species level is really not possible with ITS sequence information alone. The use of classical mycological methodologies come in handy in helping with fungal species identification and this is not to be ignored.

## 3. Novel Endophytes

The opportunity to find novel endophytic fungi at the genus or species level can be done if one’s attention is properly directed. Many of the frequently appearing endophytic fungi have their counterpart relatives existing as plant pathogens. This is especially true of the genus Fusarium which is often isolated from numerous plant species as pathogens but it seems that they are even more common as endophytes. Most interesting however, are reports of truly novel fungi as endophytes that represent novel fungal genera. Such reports require all of the work necessary to list a valid new genus/species. This necessitates getting a pure culture, and then doing the comparative taxonomic work with other related fungi at all levels including structural, developmental, and molecular as previously mentioned. Depositing the organism in a national, state or well-maintained university culture collection is also a requirement and one that is critically important. Novel endophytes can best be found in endemic plant species which are numerous in certain locations especially those hosting great biodiversity. Usually these places exist in temperate or tropical rainforests and maybe remote.

**Figure 1:**
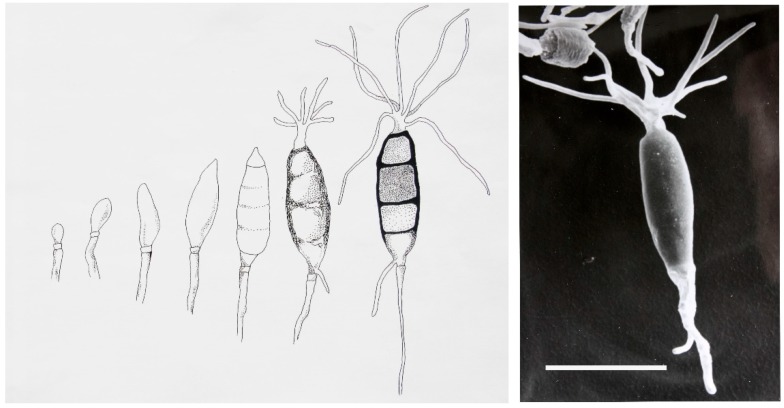
Development of the conidiospore of *Seimatoanlerium tepuiense* a novel endophyte discovered on a tepui in the Brazil, Venezuela, Guyana area of South America. The actual spore is shown to the right as a scanning electron micrograph. The bar is equivalent to 20 microns.

To illustrate the point of how to approach the discovery of novel endophytes I have shown below several examples of how we have solved the issue of biological discovery by visiting far reaching places in the world to find novel fungal genera. For instance, the fungus—*Seimatoantlerium tepuiense* was discovered as an endophyte of *Maguireothamnus speciosus* on top of the Roraima tepui on the border junction of Guyana, Brazil and Venezuela. The area is very remote and the host plant is endemic to this location and only two other small locations in tropical America. To get to this spot required the use of a rented helicopter and some knowledge of the local flora. The conidia of this endophyte resemble those of the commonly isolated endophyte-*Pestalotiopsis macrospora* however, the condiospore develops an appendage that is much more complex than the helicopter-like appendage in *P. macrospora* (Figure 1). In this case it forms an elk antler-like appendage and this structure easily dehisces from the spore (Figure 1) [17]. Presumably this allows for easy distribution by birds visiting the N.E. South American tepui plateaus since the spores can easily become attached to the fine structures of their feathers and eventually fall as the appendage becomes detached from the spore [17]. The fungus also makes small amounts of the anticancer drug-taxol. Thus, in this case not only was novel taxonomy discovered but so too was a product of extreme medical importance also found quite to our surprise.

One of the most important novel endophytes that we have ever isolated is *Muscodor albus*. This whitish, non-spore producing endophyte was originally obtained from a cinnamon tree (*Cinnamon zeylanicum*) in Hondouras [18]. This endophyte is relatively slow growing on potato dextrose agar and it makes a plethora of volatile organic compounds that possess strong antimicrobial activities [19]. In fact, the volatiles in split plate agar assays are not only inhibitory to other microbes but in most cases are lethal [19]. This makes for a likely prospect that the fungus itself might be useful as a biological control measure. In fact, in November of 2016, the US—Environmental Protection Agency approved the use of this organism for applications in US agriculture. Field testing by Marrone Bioinnovations Co. in Davis, California, has shown efficacy of *M. albus* as a pre-plant soil additive to effectively eliminate root rot causing pathogens in fields of strawberry and celery. Numerous other applications of this fungus to control disease problems in various crops has been demonstrated by Mercier and his group [20,21]. The fungus has the potential to replace the use of methyl bromide as a soil sterilant. It is the first broadly active antimicrobial producing endophyte to officially be on the market in 2017.

**Figure 2:**
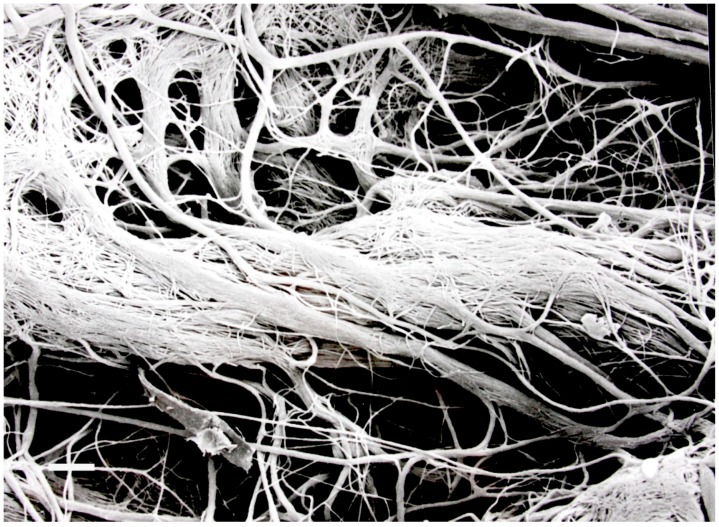
The convoluted and interwoven hyphae of *Muscodor albus*, a volatile antibiotic producing fungus. The white bar is equivalent to 5 microns.

Since its discovery almost twenty years ago many other species of this genus have also been described and now number over twenty. All members of this group have several things in common—(1) they have close homology at the ITS r DNA level [22], (2) they each produce bioactive volatile substances and (3) they are all producing a sterile (non-spore) producing mycelium that is commonly convoluted, interwoven, and sometimes possess unique hyphal projections (Figure 2) [23]. One interesting example of a novel species of this fungus is *M. vitigenus,* which was obtained from *Paullinia paullinoides* in the Peruvian Amazon, and it makes only one volatile which is naphthalene, an insecticide [24]. Cultures of *M. vitigenus* were extremely active in repelling insects in Y-tube testing experiments [25].

Another unique species is *Muscodor crispans* whose volatile composition is extremely antimicrobial, and the majority of its active compounds are on the FDA-GRAS list, that is Generally Recognized As Safe [26,27]. Thus, the bioactive volatiles can be either synthesized or made individually by fermentation processes using other microbes to make large quantities of the product in a liquid state (N. Gandhi unpublished, 2018). It has been learned that all of these volatile compounds in the fungal gas mixture are not needed for biological activity, but certain ones act in a synergistic manner to cause microbial inhibition and death of target microbes. Those that have such activity have been termed synergistans [28]. The products of *M. crispans* (Jeneil Biotech name of Flavorzon) at the 1% level has antimicrobial activity that parallels that of Clorox or quaternary ammonium [29]. This makes the solution usable on biological or industrial surfaces that may be contaminated or have the potential to be infected by or contaminated with organisms such as *Salmonella* sp. [29]. Since it is a safe product it has been used to decontaminate rooms where food-grade work is performed. Presently, another version of the Flavorzon formula is being tested for the prevention of decay in post-harvest fruits/vegetables. Still another version of the fungus gas formula is now being used to enhance the shelf life of certain products such as soy milk and is in the market place.

It turns out that each of these *Muscodor* spp. that were unique species, was patented by us at the time that they were discovered. Likewise, claims were made for the volatile chemistry that was discovered in each organism. In each case the US and many International patent offices approved the applications. It seems as if this intellectual property approach represents the best mechanism to make the discovery process useful to humankind. It is something that needs to be considered as one does all of the work to make important, valuable, and useful discoveries. Without intellectual property, a discovery such as the ones related to Muscodor are interesting but are rendered useless unless protection is sought and product development is pursued. Ultimately, the general public benefits from all of the efforts made in work of this type.

As a side light and most pleasing to me is the efforts of a group of Indian scientists who have recently described *M. strobelii* from *Cinnamon zeylanicum*, the same species from which the original isolate of muscodor was obtained, only in this case the host tree was growing in southern India, which is most likely the same source area for the Honduran tree mentioned above. The organism was unique from other species in this genus by virtue of its production of very unique volatiles, its antimicrobial activity and its unique hyphal structure [30].

## 4. Bioactive Products of Endophytes and Their Utility

**Figure 3:**
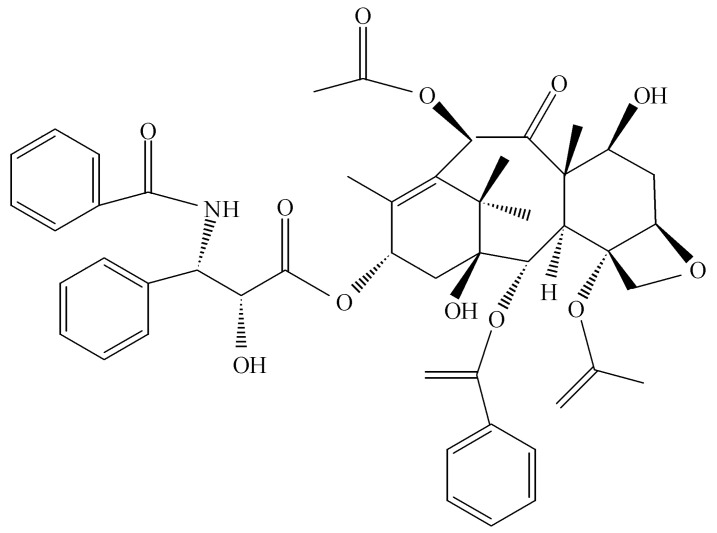
The structure of taxol.

Most work on the secondary products of endophytes arose in the 1980s with a focus on the toxic compounds associated with diseases in livestock. These symptoms were associated with fescue grass that was infected with *Neotyphodium* sp. that produces toxic metabolites causing abortion and even death in animals grazing especially on this grass species [31]. Endophytes, during that era, received notoriety and the greatest interest in them was for the damage that they caused in livestock. The broader picture of their benefit to plants and mankind was not evident until later. Then with the advent of the discovery of *Taxomyces andreanae* in Montana in a yew tree, making the anticancer drug taxol, the concept of endophytes as a source of novel and useful drugs was introduced (Figure 3) [32,33]. At the outset, it seems only logical that such possibilities exist in nature if one assumes that the endophyte and the host plant are living in some sort of symbiotic relationship and horizontal gene exchange may occur between the symbionts. Thus, any contribution to the survival of a plant has the potential to be translated into products that also might be beneficial to mankind once isolation of the endophyte is achieved along with its biologically active products. As it turns out, taxol is a potent anti-oomyceteous compound and its mode of action on fungal pathogenic oomycetes is identical to its activity against rapidly dividing cancer cells [34]. It seems likely that taxol and its hundreds of related compounds are produced by *Taxus* sp. to protect the plant from attack by “water molds.”

In my experience, any search for a natural product (with a given bioactivity) is driven by what system of bioassays might be in place or available at the time of fermentation of the fungal culture and the purification procedures to which it may eventually be applied—bioassay guided fractionation. A good and effective bioassay can greatly assist in the eventual isolation and purification of a bioactive product by allowing an assessment of where on a TLC plate or a column eluent the product of interest may be located. To this end, over the past 20 years, a plethora of review papers have appeared on the secondary natural products of endophytes and their associated biological activities [35,36,37,38,39]. These reports nicely show that endophytes are a wonderfully unique and growing source of biologically active molecules and one must be prepared to work on virtually any class of biologically active molecules as one follows the bioactivity through the purification processes.

Thus, once an endophyte is isolated in pure culture some form of fermentation methodology is employed in order to find and characterize biologically active secondary products made by the organism. Normally, a more defined medium such as the M-I-D medium is used rather than more complex media [3]. The use of a more defined medium necessarily eliminates many medium products from the purification processes. Generally, we carry out fermentation in still culture in large flasks for a period of 2–3 weeks. Initial extraction is done with methylene chloride, or ethyl acetate or n—butanol. This step is followed by flash chromatography, then preparative thin layer chromatography and HPLC. After each step the various fractions are subjected to the biological assay that has been selected. The final and most desirable goal is to get a product that is crystalline so that the product may be subjected to X-ray crystallographic analysis. Ultimately, all other spectroscopic analytical data should be in agreement with the X-ray data.

A few of the products of endophytes on which we have worked are discussed and illustrated below. The first three products were all obtained from various endophytic isolates of *Pestalotiopsis microspora* that were found in various plants in various locations around the world. This fungus is one of the most frequently isolated endophytic fungi from tropical and temperate rainforests plants. It has been seen many times and assigned many different species names, usually solely based on the host plant from which it was isolated. Until recently little work has been done on the biological chemistry of this organism. It turns out to have a treasure trove of interesting novel and bio-active molecules. Each of the products, shown below, has a unique biological activity and the chemistry of the products is also unique. This nicely illustrates the point that one must be prepared to deal with a variety of techniques in separation science and chemical characterization when dealing with the bioactive products of endophytes.

### 4.1. Pesatcin

**Figure 4:**
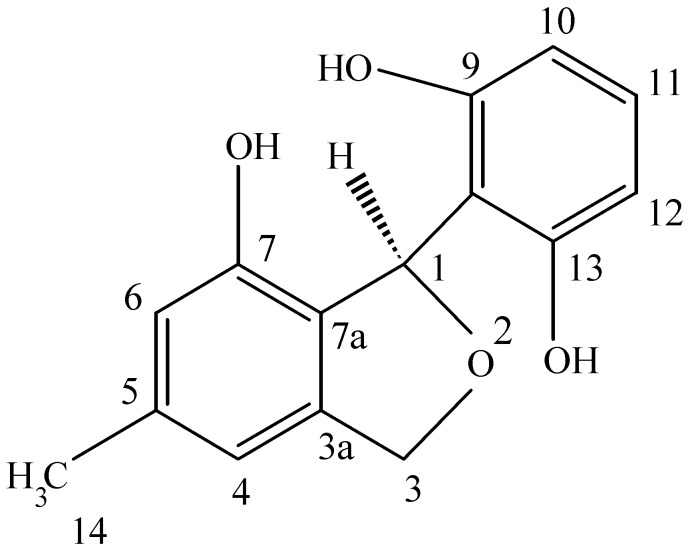
The structure of pestacin.

This novel benzofuran was obtained from a culture of *Pestalotiopsis microspora*, an endophytic fungus obtained from *Taxus wallichiana* sampled in the Himalayan foothills at which time we were engaged in looking for other endophytes making taxol and were surprised in finding an endophte making both taxol as well as two novel antioxidants. This fungal endophyte produces a new 1,3-dihydro-isobenzofuran which exhibits antioxidant activity eleven times greater than the vitamin E derivative troxol. The compound also has moderate antifungal activities [40] (Figure 4). Isolation of pestacin was achieved by extraction of culture fluid with methylene chloride followed by silica gel chromatography. Its structure was established by X-ray diffraction and ^13^C and ^1^H NMR spectroscopies. The X-ray data demonstrated that pestacin occurs naturally as a racemic mixture. Mechanisms for antioxidant activity and post-biosynthetic racemization have been proposed. Isopestacin is also produced by this endophyte and it also possesses similar bioactivities as pestacin [41].

### 4.2. Ambuic Acid

**Figure 5:**
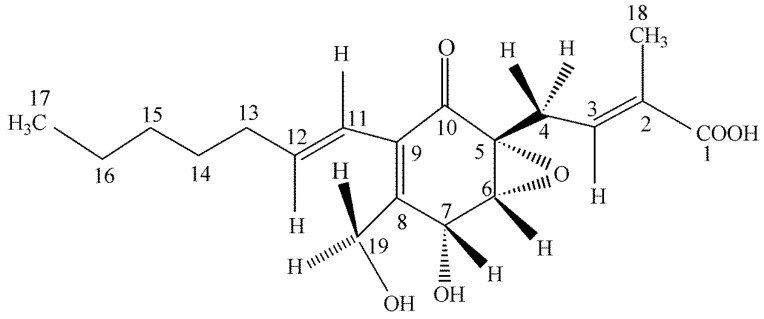
The structure of ambuic acid.

We were greatly surprised to find a highly functionalized cyclohexenone being produced by an isolate of *Pestalotiopsis microspora* as an endophyte of *Fagraea bodenii* found in the highlands of Papua New Guinea [42] (Figure 5). The compound possesses weak antifungal properties. It was first natural product to have its absolute structure established by solid state NMR methods allowing a spatial assignment to the –OH group on carbon 7 [43,44] (Figure 5). Quite surprisingly, after the initial work on the isolation and structural determination of ambuic acid, it was later learned that it is one of the best compounds known for its anti-quorum sensing activity in Gram-positive bacteria [45]. Ambuic acid inhibits the biosynthesis of the cyclic peptide quormones of *Staphylococcus aureus* and *Listeria innocua*. Ambuic acid is a lead compound in the search for anti-pathogenic drugs that target quorum sensing—mediated virulence expression of Gram positive bacteria. Once again, what initially was supposed as a weak antifungal agent turned out to have a totally unsuspected biological activity as an inhibitor of quorum sensing in bacteria and this point was not established by us but by other investigators in Japan. An assay for anti-quorum sensing activity was not originally in our repertoire of bioassays.

### 4.3. Torreyanic Acid

**Figure 6:**
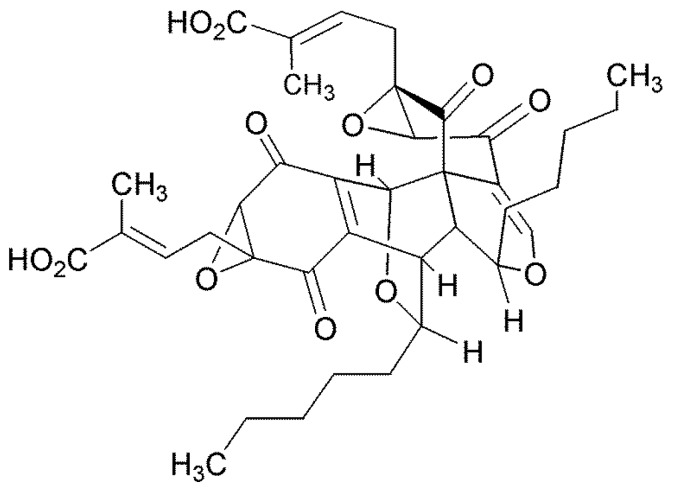
The structure of torreyanic acid.

The Florida Torreya is a rare and threatened plant and for this reason it was sampled for the presence of unusual endophytes. Torreyanic acid was isolated and characterized as a dimeric quinone obtained from the endophyte, *Pestalotiopsis microspora* originally isolated from *Torreya taxifolia* in Northern Florida [46] (Figure 6). The compound was cytotoxic against 25 different human cancer cell lines with an average IC_50_ value of 9.4 µg/mL, ranging from 3.5 (NEC) to 45 (A549) µg/mL. Torreyanic acid is 5–10 times more potent in cell lines sensitive to protein kinase C (PKC) agonists, 12-*O*-tetradecanoyl phorbol-13-acetate (TPA), and was shown to cause cell death via apoptosis. Torreyanic acid also promoted G1 arrest of G0 synchronized cells at 1–5 µg/mL levels, depending on the cell line. It has been proposed that the eukaryotic translation initiation factor EIF-4a is a potential biochemical target for the natural compound. Additionally, it has been prepared by organic synthetic techniques.

### 4.4. Colutellin A

*Colletotrichum dematium* is an endophytic fungus recovered from a *Pteromischum* sp. growing in a tropical forest in Costa Rica [4]. Strangely enough this was the only endophyte isolated from samples of this plant and this is unusual circumstance especially in dealing with tropical plant species. This fungus makes a novel peptide antimycotic, Colutellin A, with minimum inhibitory concentrations of 3.6 μg/mL (48 h) against *Botrytis cinerea* and *Sclerotinia sclerotiorum*, respectively. This peptide has a mass of 1127.70 and contains residues of Ile, Val, Ser, N-methyl-Val, and β-amino-isobutryic acid in nominal molar ratios of 3:2:1:1:1, respectively. Independent lines of evidence suggest that the peptide is cyclic and sequences of val-ileu-ser-isoleu as well as ileu-pro-val have been deduced by MS/MS as well as Edman degradation methods. Colutellin A inhibited CD4—T cell activation of IL-2 production with an IC_50_ of 167.3, whereas cyclosporine A, in the same test yielded a value of 61.8 nM. Since IL-2 production is inhibited by Colutellin A, at such a low concentration, this is an effective measure of the potential immunosuppressive activity of this compound. On the other hand, in repeated experiments, cyclosporin A at or above 8 μg/mL exhibited high levels of cytotoxicity on human peripheral blood mononuclear cells whereas, Colutellin A or DMSO alone, after 24 and 48 h of culture, exhibited no toxicity. Because of these properties Colutellin A has potential as a novel immunosuppressive drug [47].

### 4.5. Cryptocin

**Figure 7:**
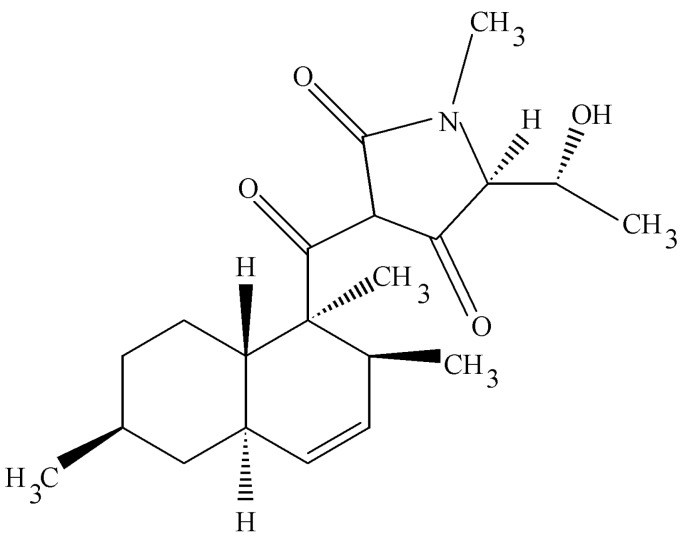
The structure of cryptocin.

*Tripterygium wilfordii* is an Asiatic plant with strong immunosuppressive properties. It was targeted for study to learn if any of its endophytes might also make the same or related biologically active molecules. This rationale for this work follows that of the fungal taxol story. Instead, one of the endophytic fungi that was isolated was a unique species of *Cryptosporiopsis*. It was unique in that its large conidiospore was segmented quite unlike other members of this group [48]. This organism possessed unusual antifungal activities that were related to two novel compounds—cryptocandin (a novel lipopeptide) and cryptocin, an unusual tetramic acid. The acid was isolated and characterized by X-ray crystallography and other spectroscopic data (Figure 7) [48]. The compound was active against many plant pathogenic fungi (<1 µg per mL) and much less impressive activity against human pathogenic fungi (>50 µg per mL). However, to be noted is that the most sensitive fungus was *Pyricularia oryzae*, the causal agent of rice blast one of the most important pathogens in the world as it relates to food production.

The search for these valuable products has just begun. Usually, in my experience, there are several initial clues that make an organism a potential candidate for a producer of novel products. If it had been isolated from a unique environment or an endemic species, if it is a slow grower on common lab media and if it is coming from a totally unique fungal genus, the chances for finding something new are greatly improved. It is not, however, uncommon to work on an organism only to find that the bioactive product already been discovered in the same or related organism by someone else. Thus, novelty is a major goal! Repeated again, once the work is completed and a novel bioactive product has been discovered, one should make an effort to get patent coverage and find an interested commercial partner in order for some aspect of the general public to directly benefit from the discovery.

## 5. Fungal Hydrocarbons and Fuels

We have relatively recently described endophytic fungi that make one or more hydrocarbons that have potential as fuels. Such products have been dubbed –mycodiesel [49]. This dramatically differs from standard yeast fermentation processes that utilizes sugars or starch to produce ethanol which is a proven and useful source of fuel, but by no means is it ideal. A number of endophytic fungi have been isolated and described that make compounds such as mono-terpenoids, alkanes, cyclohexanes, cyclopentanes, and alkyl alcohols/ketones, benzenes and polyaromatic hydrocarbons [4]. Many of these compounds are either identical to or are closely related to those specific classes of molecules that are found in diesel/gasoline [4,50]. Most importantly, these organisms make hydrocarbons while utilizing cellulose, hemicellulose and other polymers found in all plant-based agricultural wastes [51,52,53]. Endophytes are a prime source of hydrocarbon producers because they are the first microbes to begin the processes of tissue degradation, when a plant dies, to yield products with fuel–potential [4,53]. Examples of fungi that have been discovered that make fuel related hydrocarbons include—*Ascocoryne sarcoides*, *Gliocladium* sp., *Hypoxylon* sp. (*Nodulisporium* sp.), *Annulohypoxylon* sp., *Phoma* sp., *Phomopsis* sp., and *Daldinia* sp. [13,50,51,52,53,54,55]. The volatiles produced by these organisms are usually possessing antibiotic activities which conceivably contribute to their role as symbionts. Finally, it seems possible that endophytic fungi may have an additional attribute of having contributed to the formation of crude oil in the first place and experiments have been done to demonstrate this phenomenon [53].

*Muscodor albus*, as described above, was used to discover other fungi making bioactive volatiles that turned out to be hydrocarbon-like molecules. Plant tissues of carefully selected plants were placed on half plates containing 10 day old cultures of *Muscodor albus* whose gases killed most fungi other than those also making bioactive volatiles. As an example of the use of this selection technique was the sole appearance of *Gliocladium roseum* (now classified as *Ascocoryne sarcoides*) [49,54] in Petri plates (PDA) supporting the growth of *Muscodor albus* showed that this fungus was able to survive and grow in the presence of the inhibitory and lethal volatiles (VOCs) of *M. albus* [49]. Further testing of the recovered *A. sarcoides* culture revealed that its VOCs were active against other test fungi and that some of its volatile products had fuel potential [49]. Most interesting was the appearance of a series acetic acid esters of straight chained compounds including those of hexyl, heptyl, octyl, sec-octyl and decyl alcohols [49]. In addition, many other hydrocarbons were also noted in the GC/MS analyses of the VOCs of this fungus. The straight chained hydrocarbons (alcohols in the reduced form) are the backbone compounds found in all diesel fuels that we have investigated from widely differing parts of the world.

Since those observations were made it has become increasingly evident that many other endophytic fungi making volatile hydrocarbons are also resistant to the *M. albus* VOCs and their VOCs are biologically active [4]. Thus, this one selection technique has the potential of eliminating 80–90% of endophytic microbes that probably would not be of interest relative to VOC production since they would succumb to the VOCs of *M. albus* while growing out of the plant tissues on the Petri plates. This technique has merit if one does not wish to deal with a plethora of microbes that may not be of interest and waste time doing GC/MS analyses on organisms that would not be of interest. Nevertheless, it is to be noted that some important hydrocarbon producing fungi are sensitive to the VOCs of *M. albus* and would have been missed if this technique had been exclusively used for selection [55].They can be usually selected on the basis of the biological activity of their VOCs.

**Figure 8:**
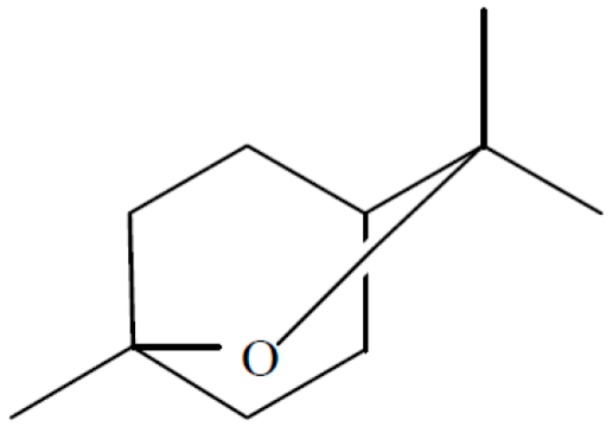
The structure of 1,8-cineole.

An endophytic fungal strain of *Hypoxylon* sp. was isolated from *Persea indica* an evergreen tree native to the Canary Islands where it grows not in abundance but is found on several islands including Tenerife in the Laurisilva [13]. This organism was isolated in its imperfect stage as *Nodulisporium* sp. from a small stem in the crown of the tree and it readily grows in the presence of the VOCs of *M. albus* which should facilitate its isolation from other plant sources. When grown on PDA—Petri plates, the VOCs produced by this fungus were primarily 1,8-cineole; 1-methyl-1,4-cyclohexadiene, and (+)-.alpha.-methylene-.alpha.-fenchocamphorone (Figure 8). Not only these but many of the compounds made by this organism are of interest because of their high energy densities and thus the potential they might have as Mycodiesel fuels [13].

Six-day-old cultures of *Hypoxylon* sp. (imperfect stage—*Nodulisporium* sp.) displayed maximal VOC-antimicrobial activity against *Botrytis cinerea*, *Phytophthora cinnamomi*, *Cercospora beticola*, and *Sclerotinia sclerotiorum* suggesting that the VOCs may play some role in the biology of the fungus and its survival in its host plant. In fact, this discovery has implications in developing methodology for strain improvement via mutation/selection techniques [13]. Media containing starch-or sugar related substrates best supported VOC production by the fungus. Continuous direct on-line quantification of VOCs was measured by proton transfer mass spectrometry (PTR-MS) covering a 12 day range with optimum VOC production occurring at 6 days at 145 ppmv with a rate of production of 7.65 ppmv/h [13]. The production of 1,8-cineole from a fungal source is of significant interest given the fact that this compound does not have any previously known biological sources aside from plant tissue, and has thus far limited the compound’s availability for fuel purposes (Figure 8).

In a related *Hypoxylon* sp. a specific cineole synthase has been identified along with 11 new terpene synthase genes [56]. The discovery is an important first step in identifying a complete fungal pathway for the synthesis of 1,8-cineole and related monoterpenes. Currently, the yields of these terpenes and other hydrocarbons from fungal cultures does not allow for the production of these compounds at an economical level. These problems may eventually be overcome with conventional mutagenseis techniques and or genetic manulipation combined with complete sequencing and annotation of important fungal genomes such as *Ascocoryne sarcoides,* which is the best annotated endophyte thus far studied [57]. In combination with these approaches it will be necessary to develop non-conventional fermentation methods which may employ novel solid state methods allowing for a constant yield of volatile products via an airsteam and appropiate gas traps (Strobel unpublished, 2018).

Furthermore, as a practical matter cineole has been examined in multiple ways for its utility as a fuel. Studies on 1,8-cineole have shown prevention of phase separation when used as an additive in ethanol-gasoline fuel blends [58]. Furthermore, when fuels comprised of a gasoline/eucalyptus oil mixture (with 1,8-cineole as the major fuel component up to 80% or more) there was an improved octane number and reduced carbon monoxide exhaust [59]. Thus, 1,8-cineole is a worthy target molecule for study and scale up and it has amazing potential for replacing fossil-based hydrocarbons as a fuel additive.

Finally, it is to be noted that to do the best work on fungal volatiles does require the use of some unique methods and techniques in qualitative and quantitative analysis of these gaseous compounds. Standard practice is the use of the SPME fiber trapping technique combined with GC/MS to acquire an idea of the compounds being produced. The advent of PTR/MS has permitted the evaluation of on line-real time analysis and quantification of fungal gas production and now PTR/TOF-MS allows for this plus qualitative analysis of the gas stream [4]. Gas trapping in stainless steel columns as well as PTR /MS in line with a platinum catalysis will permit a total organic volatile compound production analysis—again in real time [60].

## 6. Control of Secondary Product Formation in Fungi

In many cases, once a product has been isolated and identified, the fungus may become attenuated in the production of the wanted compound (s). This is an event that has happened at least 50% of the time in my experience and can cause concern and self-doubt in the mind of the investigator. This is best exemplified by the attenuation of helminthosporoside (a plant host specific toxin) produced by the plant pathogen-*Helminthosporium sacchari*. When the organism was continuously cultured and transferred on a semisynthetic medium the production of this fungal toxin totally ceased [61]. However, when amounts of leaf exudate—concentrate were placed into the cultural medium, toxin production fully resumed. It turned out that several secondary products of sugarcane metabolism were responsible for the regulation of toxin production by this pathogenic fungus. One of these compounds was isolated and characterized as serinol (primary alcohol of serine) and the other was a sugarcane lipid-like compound [62]. It seems that the best place to look for answers to problems surrounding attenuation of secondary product formation in endophytic or plant pathogenic fungi is the host itself.

Similar events happened during the course of the discovery of taxol in an endophytic *Periconia* sp. which was isolated from *Torreya grandifolia* (a relative of yew that does not synthesize taxol) in China. This fungus, not previously known as a tree endophyte, was isolated from the inner bark of a small lower limb. When freshly isolated from the tree and placed in a semi-synthetic medium, the fungus produced readily detectable quantities of the anticancer drug taxol [63]. The production of taxol by *Periconia* sp. was demonstrated unequivocally via spectroscopic and immunological methods [63]. However, successive transfers of the fungus on a semi-synthetic medium resulted in the gradual attenuation of taxol production until minimal amounts of it were produced even though fungal growth was relatively unaffected. Several compounds, known previously as activators of microbial metabolism, including serinol, *p*-hydroxybenzoic acid, and a mixture of phenolic acids, were capable of fully or partially restoring taxol production to otherwise taxol-attenuated cultures. The compound with the most impressive ability to activate taxol production was benzoic acid at 0.01 mM. To our knowledge, benzoic acid is not a direct taxol precursor [63].

The fungal fuel producing fungus—*Nodulisporium* sp. also falls into the category of an organism that will attenuate secondary product formation. It appears as if there are factors controlling 1,8-cineole and other hydrocarbon molecule production in this fungus based on the observation that serial transfer of the fungus on PDA resulted in a dramatic reduction of VOC production including 1,8-cineole [64]. Subsequently, when the attenuated organism was placed on certain plant parts and plant extracts the VOC production resumed to normal as measured by PTR-MS [64]. *Nodulisporium* sp. (Ti-13) in question had been isolated as an endophyte from *Cassia fistula* in Thailand. The fungus produces a spectrum of volatile organic compounds (VOCs) that includes ethanol, acetaldehyde, and 1,8-cineole as major components [64]. Initial observations of the fungal isolate suggested that reversible attenuation of the organism via removal from the host and at least 5 successive transfers in pure culture resulted in a 50% decrease in cineole production unrelated to an overall alteration in fungal growth. A compound (CPM_1_) was obtained from *Betula pendula* (silver birch) that increases the production of 1,8-cineole by an attenuated Ti-13 strain to its original level as measured by a novel bioassay method employing a 1,8-cineole sensitive fungus-*Sclerotinia sclerotiorum* [64]. The host plant *Cassia fistula* also produces similar compounds possessing this activity. Bioactivity assays with structurally similar compounds such as ferulic acid, gallic acid and others suggested that the CPM_1_ does not to act as a simple precursor to the biosynthesis of 1,8-cineole. Nuclear magnetic resonance spectroscopy and high-performance liquid chromatography electrospray ionization mass spectrometry indicated that the CPM_1_ is a para-substituted benzene with alkyl and carboxyl substituents [64]. The VOCs of Ti-13, especially 1,8-cineole, have potential applications in the industrial, fuel, and medical fields.

These observations are suggestive of one of more regulatory mechanisms involved in hydrocarbon production that seems to be influenced by the host plant. Obviously, these considerations on the regulation of secondary product formation will be vital in making hydrocarbon—based fuel production from fungi a reality in the future. The work also has relevance to literally any plant microbe that is under the regulation of secondary product formation by host-related substances.

## 7. Endophytes and Epigenetics

We have recently learned that the regulation of the production of fuel –like compounds in fungi is under epigenetic control. *Hypoxylon* sp. (*Nodulisporium* sp.) is of interest because of its ability to make hydrocarbon-like compounds that may serve as fuels. Thus, experimental work was undertaken to affect the gene expression of this organism through compounds known to act as epigenetic modifiers and explore the possibility of the appearance of new products and/or the increased or decreased production of other VOCs already known from this organism [65]. For instance, it is realized that the complete analyses of fungal genomes in recent times has indicated that many putative biosynthetic gene clusters are located in the distal regions of the chromosomes and exist in a heterochromatin state with the constitutive genes often transcriptionally controlled by epigenetic regulation such as histone deacetylation and DNA methylation [66,67]. Several studies have demonstrated that the inhibition of histone deacetylase activity, through gene disruption or use of epigenetic modulators, leads to the transcriptional activation of gene clusters resulting in enhanced production of secondary metabolites [66,67]. Fungi treated with DNA methyl transferase- and histone deacetylase inhibitors exhibited natural product profiles with enhanced chemical diversity demonstrating that the small-molecule epigenetic modifiers are effective tools for rationally controlling the native expression of fungal biosynthetic pathways and generating biomolecules not previously associated with the organism [66,67]. Thus, the fungus was exposed to the epigenetic modulators- histone deacetylase- and a DNA methyltransferase inhibitor -SAHA (suberoylanilide hydroxamic acid) as well as 5-azacytidine (AZA), respectively. Subsequently, the organism displayed striking cultural changes including variations in pigmentation, growth rates and odor, in addition to significant differences in the bioactivities of its VOCs [65]. Analyses (GC/MS) of the VOCs produced by the modulated fungus showed considerable variation with the emergence of several compounds not previously observed in this fungus, particularly an array of tentatively identified terpenes such as ∞-thujene, sabinene, δ-4-carene, γ-terpinene, ∞-terpinolene and β-selinene, in addition to several primary and secondary alkanes, alkenes, organic acids and derivatives of benzene. The spectrum of identifiable compounds, in this study, was greatly enhanced by virtue of the organism being altered with the epigenetic modulators [65]. It is likely that the same phenomenon would occur with other fungi but the result would be an entirely different set of secondary products.

## 8. Biology of Endophytes and Their Ultimate Utility

This review has considered only a limited range of topics concerning endophytes. Their vastness and diversity literally covers the entire planet since they are associated with all macroscopic plant life forms. Only a small portion of their range and diversity has been sampled with entire vast reaches of the world never having been sampled at all. This would include areas of central Africa, the lower reaches of the Amazon, many area of the rainforests of South Eastern Asia, as well as the expansive forests of Russia and central Asia. In addition to these sources are the macro algae of the world’s oceans along with an abundance of other plant forms such as the ferns, mosses, lichens that also need attention. Only about 1% of the world’s botanical sources of endophytes have ever sampled and incompletely at that since only a limb or leaf samples were the subject of study. It is well known that less accessible roots, fruits and the inner tissues of the stem each contains its own endophytic assemblage. The patterns of abundance and diversity of these organisms then becomes an issue for study as per Arnold et al. [68]. The biology of endophytes is also influenced by leaf or plant age, canopy cover, seasonal factors and even the presence of other endophytes [69].

Most interesting are observations that suggest that endophytes may play some role in the protection of the host plant from plant pathogens or other agents of destruction including insects and herbivores [11]. This brings into focus the potential roles of endophytes in enabling the host plant to be best fit for its survival and the potential that exists for harnessing these organisms for the benefit of agriculture [11,70]. Thus, managing the plant microbiome is a goal of several companies that wish to take advantage of the role that endophytes may play in the health, fitness, growth and development of the plant in its environment. Some of these are Indigo (Cambridge, MA, USA), Bayer/Ginko Bioworks (West Sacramento, CA, USA) and MBI (Davis, CA, USA). Many in the world of agricultural business would agree that managing a living organism as a product can be fraught with some problems. These are usually lack of reliability and consistency of outcomes when placing the live product on the market.

Some guidance for the development, and testing endophytes for eventual commercialization has recently been presented [71]. This work was done because of the problems mentioned above. Murphy et al. [71] began their work on barley by finding candidate source endophytes in a wild relative of *Hordeum mirnum* followed by testing in a carefully controlled environment and finally doing multi-year field testing. In their hands this approach was successful. It is to be strongly stressed in that what is generally missing in most studies of endophytes of crop plants is exactly what was avoided by Murphy et al. [71]. The organisms of interest to them were recovered by an extensive search and description of the endophytic flora of wild host plants growing in or near the center of origin of that species. It is entirely likely that that most plants serving mankind as food sources have long since lost their complement of normal flora as a result of movement from their centers of origin, intensive genetic selection techniques dealing only with the seeds or fruits of the plant and exposure of the crop to a myriad of pesticides. Evidence for this concept has been demonstrated in wild wheat related species vs. *Triticum aestivum* [72]. The wild types relatives of wheat contain a plethora of taxonomically diverse fungal endophytes that are not found in modern wheat. It turns out that most wild or native food source plants have yet to have their microflora studied. It behooves us to find and carefully preserve all natural sites that harbor wild relatives of mankind’s major and minor food plants not only for the genetic diversity that they may hold but also for the unique microflora of these plants that may exist in such places.

## 9. Endophyte Biology as a Platform for Teaching Undergraduate Students

Finally, it should be mentioned that the concepts and techniques involved in isolating and characterizing endophytes and studying their biology represents an excellent opportunity for independent undergraduate participation. Several studies have demonstrated that an independent research experience is the best way to get undergraduates excited about a career in science [73]. In fact, the vast majority (around 80%) of the science faculties in US universities had an undergraduate research experience during their college careers. In this case a student can do her/his own collecting, plant identification, finding GPS coordinates and bringing the samples to the lab for further work. Endophyte isolation, microbial identification using classical methods and modern molecular methods offer a student a real opportunity to acquire independent project ownership which is a great way to encourage progress on a project. Ultimately, the student will have the possibility to develop novel bioassays for endophyte products or learn a myriad of other techniques involving chemistry, molecular biology, statistical methods and spectroscopic methods among others as applied to the microbes that were isolated. The student may have an opportunity to publish her/his work and possibly be involved in the patenting process. In fact, in this review I have cited many papers from my lab as the reference list to this paper. The names of Mitchell, Daisy, Tomsheck, Schaible, Mends and Nigg had all worked as undergraduate students in my laboratory and the majority of these students were women. Each of these students has had one or more senior authored papers from one or more individual research projects and their names appear in the Reference section of this paper [13,24,25,26,27,28,55]. The concept of giving undergraduate students and opportunity to work on endophyte projects has been as rewarding for them as it has for me.
